# Web-Based Acceptance and Commitment Therapy Tobacco Cessation Program for Veterans With Mental Health Disorders: Adaptation and Usability Testing

**DOI:** 10.2196/75394

**Published:** 2026-02-26

**Authors:** Megan M Kelly, Abigail Dempsey, Victoria Ameral, Beth Ann Petrakis, Erin D Reilly, Karen Quigley, Jonathan B Bricker, Jaimee L Heffner

**Affiliations:** 1 VISN 1 Mental Illness Research, Education, and Clinical Center VA Bedford Healthcare System Bedford, MA United States; 2 Department of Psychiatry and Behavioral Sciences University of Massachusetts Chan Medical School Worcester, MA United States; 3 Department of Psychology Purdue University Northwest Hammond, IN United States; 4 Department of Psychology College of Science Northeastern University Boston, MA United States; 5 Division of Public Health Sciences Fred Hutch Cancer Center Seattle, WA United States; 6 Department of Psychology University of Washington Seattle, WA United States

**Keywords:** tobacco cessation, smoking, veterans, mental health disorders, acceptance, mindfulness, acceptance and commitment therapy

## Abstract

**Background:**

US veterans with mental health disorders have high rates of smoking and low rates of smoking cessation.

**Objective:**

This study aims to focus on an adaptation of a web-based acceptance and commitment therapy (ACT) tobacco cessation intervention (Vet WebQuit) for veterans with mental health disorders who use tobacco and used a qualitative approach to test its usability (n=16).

**Methods:**

Participants were asked to walk through the site during laboratory-based usability testing and “think aloud” about the features of the intervention. A trained facilitator used semistructured interview questions to assess participants’ experiences with Vet WebQuit and obtain feedback on their impressions of the site. Qualitative analyses identified themes regarding participants’ experiences with the intervention, usability concerns, and recommendations for improving Vet WebQuit.

**Results:**

Overall, veterans found that the Vet WebQuit layout was simple and easy to navigate and use. Veterans reported that several features of the program were useful, including the quit plan, identification of triggers, content that targets mental health concerns (eg, dealing with anger), information on the health effects of smoking, tools for managing triggers (eg, urge surfing), and involving others in their quit plan. Veterans reported that particular features of the ACT approach for tobacco cessation were appealing to them, including the distinction between internal and external smoking triggers, the inclusion of the serenity prayer, and mindfulness exercises, which they could use as a tool reduce the intensity of cravings. Veterans reported wanting more information on the health aspects of smoking (ie, effects on breathing and lung capacity) as a way to motivate them to quit smoking. In addition, they suggested targeting specific mental health concerns that serve as triggers for smoking, including nightmares, boredom, and social isolation.

**Conclusions:**

Overall, results from this project identified important elements of ACT digital tobacco cessation interventions for veterans with mental health disorders.

## Introduction

Smoking is significantly more common among veterans relative to nonveterans [1], and veterans with co-occurring mental health diagnoses carry an even heavier burden, with high rates of smoking (32%-68% vs 15% in the general population) and low lifetime quit rates (17-33% vs 43% in the general population) [2-4]. Individuals with mental health disorders have less successful quit attempts [5-7], increasing the chances of experiencing serious medical consequences, including myocardial infarctions, stroke, and cancer [8] and an average of 25 years of potential life lost [9]. Challenges with quitting smoking are often directly related to mental health symptoms [6,7,10,11]. People with tobacco use and co-occurring mental health disorders frequently report smoking to manage their psychiatric symptoms [12-14]. This is a critical challenge, as smoking does reduce anxiety and improve mood in the short-term [15], but with deleterious long-term effects on mental health [16,17] and worse outcomes of treatment for co-occurring conditions [18,19]. Further, mental health symptom severity predicts heavier smoking for veterans with common co-occurring conditions, including posttraumatic stress disorder (PTSD) [20]. Despite the role of psychiatric symptoms as a key barrier to quitting [10,11], they are not a major focus of most current treatments.

Standard tobacco cessation treatments typically use a cognitive-behavioral therapy (CBT) approach [21], which teaches people to avoid tobacco triggers, change routines, and alter thoughts about smoking. These treatments often do not focus on the mental health concerns that make it difficult for veterans with mental health disorders to quit, and existing population-level tobacco treatments (eg, the Veterans Healthcare Administration [VHA] National Quitline) for veterans are provided by cessation counselors for whom mental health symptoms are outside their scope of competence. The most widely studied tobacco cessation treatment for veterans with mental health disorders showed that integrating standard tobacco cessation techniques into PTSD treatment modestly improved cessation outcomes compared to traditional treatment (14% vs 8% at 3 months) [22]. Another trial of concurrent PTSD and standard tobacco treatment found that participants significantly decreased their cigarette use during treatment, but had not quit smoking [23].

Acceptance and commitment therapy (ACT) for smoking cessation is an evidence-based approach with great potential to help veterans with co-occurring mental health disorders. ACT fosters psychological flexibility (ie, the ability to act in the service of personally meaningful values while remaining open to internal experiences [eg, thoughts, feelings, and sensations]). ACT does this by targeting 6 interrelated psychological processes: acceptance, cognitive defusion (ie, learning to see thoughts as passing mental events), present-moment awareness, self-as-context (ie, cultivating a perspective of self as the observer of experiences rather than being defined by them), values clarification, and committed action [24]. ACT uses mindfulness (ie, focus on the present moment with a nonjudgmental attitude) to help individuals accept aversive internal experiences (eg, smoking cravings and nicotine withdrawal symptoms), rather than trying to control or change them. Empirical support for ACT for smoking cessation comes from several randomized controlled trials demonstrating the efficacy of ACT versus pharmacotherapy and CBT-based treatments [25,26].

There is increasing interest in adapting these interventions for people who smoke and have co-occurring psychiatric disorders due to ACT’s demonstrated efficacy for both smoking [26-31] and mental health disorders [24,32-37]. A targeted ACT approach offers advantages over traditional tobacco treatment, which typically uses a CBT approach [21]. One major issue with a CBT approach to tobacco cessation is that avoiding triggers may not be possible, particularly “internal” triggers, which include affective, physical (eg, pain), and cognitive triggers for smoking. In addition, these treatments often do not focus on the mental health obstacles to quitting. In contrast, ACT tobacco cessation treatment helps individuals pursue what is important to them (ie, quitting smoking) by increasing willingness to experience affective, physical, and cognitive triggers for smoking (eg, mental health symptoms).

A novel, web-based delivery method increases the availability of needed tobacco treatment and addresses key barriers to smoking cessation care for veterans. First, lack of clinician training in tobacco treatment is a major barrier to the accessibility of treatment in mental health care settings [38-40]. Self-guided, web-based interventions provide a referral option that requires no knowledge of tobacco treatment. Second, veterans report limited appointment hours and long waitlists as key barriers to tobacco cessation treatments [41], both of which are addressed by the accessibility and flexibility of web-based interventions. Additional benefits include the cost-effectiveness and ease of disseminating web-based interventions [42]. Finally, web-based treatments have high potential reach, as 76-80% of veterans use the internet at home or on mobile devices [43,44] (somewhat lower for rural veterans at 53% [45]), including 70% of veterans with serious mental illness (SMI) [38], and 56-76% of veterans are interested in using VHA health-related programs [43,44].

There is good evidence to support a web-based ACT intervention for smoking cessation, WebQuit, with a nonveteran population [39,40]. In this first study, participants were randomized to either the ACT intervention (WebQuit; n=111) or the National Cancer Institute’s Smokefree.gov web page, the current standard for web-based smoking cessation interventions (n=111). WebQuit [40] was more efficacious than the standard web-based smoking cessation intervention for people who smoke in the general population (23% vs 10% at 3 months). A subsequent larger randomized controlled trial also showed that WebQuit was as effective as CBT (24% vs 26%) [39], and WebQuit was highly acceptable to people who smoke with clinically significant symptoms of PTSD [46].

Building on this promising evidence, we developed Vet WebQuit, a targeted intervention that has strong potential to improve the efficacy and accessibility of tobacco treatment for veterans with mental health disorders. As part of the development process, our team conducted work to examine the usability of this adaptation, or the extent to which a website can be easily navigated and understood by users. Usability is a critical outcome of a web-based program development process, as low usability can undermine engagement with the intervention [47] as well as its potential efficacy for smoking cessation [48]. While the original version of WebQuit underwent usability testing as part of its development, additional testing was needed to examine the veteran-specific elements of the Vet WebQuit program. This was particularly crucial given our focus on co-occurring mental health conditions, as there is evidence of wide-ranging usability problems in existing smoking cessation websites for individuals with SMI [49]. Further, some mental health disorders (eg, psychotic disorders and PTSD) are associated with neurocognitive impairments, including executive functioning, attention, and verbal memory and learning [50-52], all of which could influence usability. This study evaluated interview data from veterans about the usability of the Vet WebQuit website, focused on key usability areas [53] to identify the most and least favored aspects of the site, review usability challenges, gather feedback on ideas for improving the site’s usability, and understand overall satisfaction with the site.

## Methods

### Recruitment

Inclusion criteria were (1) current *DSM-5* (*Diagnostic and Statistical Manual of Mental Disorders* [Fifth Edition]) mental health disorder, (2) regular smoking for at least 3 years, (3) currently smoking at least 5 cigarettes per day, (4) competent to provide written informed consent, (5) at least weekly access to a high-speed internet connection, (6) willing and able to read in English, and (7) age 18 years and older. Exclusion criteria included (1) current or recent (within 1 month of study entry) moderate or severe *DSM-5* alcohol or drug use disorder, (2) cognitive impairment that would interfere with participation, (3) suicidal behavior or severe suicidal ideation within the past 3 months, and (4) a psychiatric hospitalization within the past month.

Participants were first prescreened by phone, then invited to attend a more comprehensive virtual screening. At the beginning of the screening, participants were provided with detailed information about the study, and informed consent was obtained. Following informed consent, participants completed study assessments, including the *Structured Clinical Interview for DSM-5 Research Version (SCID-5-RV)* [54], a semistructured interview guide for making mental health diagnoses, and the Smoking History Questionnaire [55], which is a measure of smoking history and patterns. Participants who remained eligible following the virtual screening were enrolled in the usability testing procedure. No participants were ruled out based on virtual screening (n=20), 4 were lost to follow-up after screening, before completing usability testing, for a total sample of 16. Participant numbers in the Results section will range from 1 to 20 based on the number of participants who were originally consented. We chose to interview 16 veterans since the average number of participants needed to reach saturation for usability testing is 10 (SD 2) [56], and we added 4 more interviews beyond the upper limit of the recommendation to ensure saturation. All eligible participants were provided with smoking cessation resources after the usability testing procedure.

### Vet WebQuit Program

The Vet WebQuit program is a veteran-focused adaptation of the evidence-based WebQuit program. The goals of the intervention are to (1) develop acceptance*-*related skills for dealing with internal smoking cues while (2) enhancing commitment to quit smoking and making improvements in life functioning [31]. Participants are encouraged to make behavioral choices guided by valued life goals, rather than modifying or alleviating certain sensations, emotions, and thoughts. To this end, the intervention focuses on ACT’s core interdependent processes: acceptance and commitment*.* In ACT for smoking cessation, acceptance refers to allowing oneself to have, without defense, the sensations, emotions, and thoughts that cue smoking [27,29-31]. The process of commitment in ACT means committing to taking actions to quit smoking, even in the presence of smoking cues*.*

The original WebQuit program has 4 parts. Part 1, make a plan, allows users to develop a personalized quit plan, consider smoking triggers, learn about cessation medications, and upload a photo of what inspires them to quit (ACT processes: values and committed action). Part 2, be aware, contains ACT exercises to illustrate the problems with trying to control thoughts, feelings, and physical sensations rather than allowing them to come and go (ACT process: acceptance). Part 3, Be Willing, contains a series of ACT exercises designed to help the user practice by allowing uncomfortable thoughts, feelings, and physical sensations to be present, without trying to change them by smoking or using some other means of control (ACT processes: acceptance, being present, and cognitive defusion). Part 4, Be Inspired, contains ACT exercises to connect deeply held values to the user’s motivation for quitting and to exercise self-compassion in response to difficult experiences, including smoking lapses (ACT processes: values and self-as-context). The program also prompts users to track smoking, use of cessation medications, and practice of ACT skills. To increase interactivity and personalization, tracking results are displayed graphically as progress reports on the home screen, along with the user’s inspiration for quitting and badges earned for program use.

Vet WebQuit is built on the content of the original WebQuit program by incorporating ACT exercises that specifically address challenges to quitting for veterans with mental health disorders. This includes specific content on managing common co-occurring emotional challenges for veterans (ie, anxiety, anger, trauma, and substance use) as triggers for tobacco use. Veterans are encouraged to identify their values and activities that they can accomplish to help them quit smoking, improve their management of psychiatric symptoms, and increase their quality of life. The new content (Table 1) also specifically addresses the culture among veterans that encourages smoking and asks veterans to choose to quit based on their values and not on social pressure to smoke (eg, mentioning that smoking often started in the military, that it was social and common, and often used as a reward). Veterans Affairs (VA) tobacco cessation resources are embedded in the adapted program (eg, VA tobacco cessation information, links to online VA resources and programs). Veteran-specific quit stories based on real veterans were included with a focus on co-occurring substance use, PTSD, and anxiety to help veterans with mental health disorders feel inspired by other veterans in their journey to stop smoking.

**Table 1:** Vet WebQuit content tailored for veterans with mental health disorders.

Purpose			Description
**New Content for Vet WebQuit**			
	Increase motivation to make life changes	Values clarification exercise to motivate veterans to quit smoking and identify other life goals (eg, social and community involvement) that are connected to values.	
	Identify new coping skills to improve functioning	More committed action exercises to help veterans identify activities to improve mood, replace smoking, and improve quality of life.	
	Reduce anxiety over making life changes	More exercises focused on increasing willingness to experience uncomfortable thoughts and feelings about quitting and making life changes for the sake of living one’s values.	
**Specific Content for Veterans with Mental Health Disorders**			
	Veteran-specific content	Imagery and exercises are tailored to veterans (eg, veteran-specific images, stories, and exercises), and address the veteran culture of smoking.	
	Embed VA^a^ resources	VA tobacco cessation resources are embedded (eg, VA tobacco cessation information, links to online VA resources and programs).	
	Target mental health symptoms as triggers	Specific content on how to manage common co-occurring symptoms for veterans who smoke (eg, anxiety and anger) as triggers for tobacco use.	

^a^VA: Veterans Affairs.

**Figure 1:**
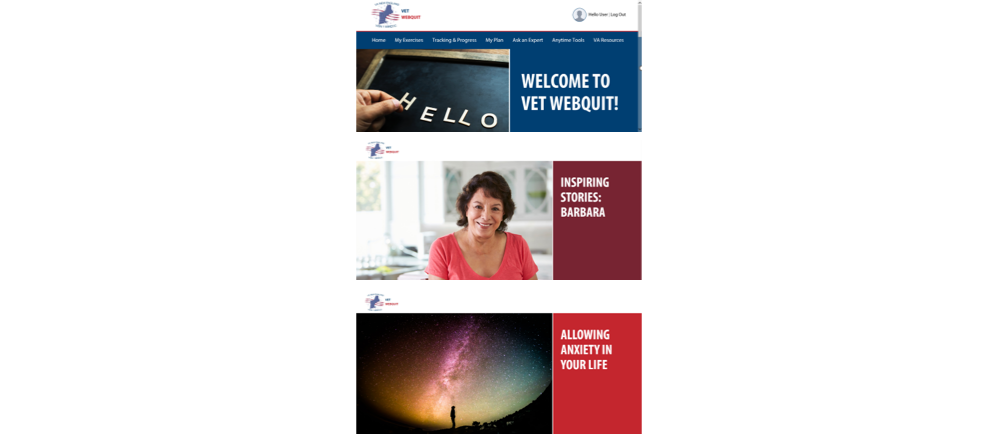
Vet WebQuit images.

Images for the program (Figure 1) were chosen by veterans based on how close they felt the image represented a particular ACT concept (eg, a person with open arms representing acceptance). In development work leading up to the creation of the Vet WebQuit program, veterans indicated that they did not prefer military-specific images (eg, helicopters and jets), but did like images of nature and the American flag. These images were embedded in the Vet WebQuit program, and the VA-approved color scheme was used throughout the program.

### Usability Testing Procedures

Eligible veterans participated virtually over VA-approved video conferencing software. Each recorded usability testing session took about 1.5-2 hours to complete. A trained facilitator used semistructured interview questions to assess participants’ experiences with Vet WebQuit and obtained feedback on impressions of the site. Participants were asked to interact with the Vet WebQuit program and complete 4 tasks: setting up a quit plan, tracking progress, viewing progress, and completing an ACT exercise. At the beginning of the usability testing session, a trained facilitator provided the following instructions:

In a few minutes I’m going to show you the Vet WebQuit website. I’m going to ask you to try things out. As you’re trying them out, think aloud—whatever comes to mind as you’re seeing the program for the first time.

Facilitators indicated that they wanted participants’ honest opinions about what they experienced. The facilitator guided participants through the usability testing procedure but did not intervene or disrupt the thinking-aloud process. The facilitator provided instructions on how to navigate to each task (ie, since tasks were spread throughout the program), but the participant was asked to navigate to the task themselves and complete it on their own.

We asked participants if they had challenges with using the site, their most and least favorite aspects of the intervention, and any recommendations they had for improving the site. We assessed Vet WebQuit’s usability along the following dimensions that are recommended for usability testing by the US government [57]: (1) intuitive design: understanding the setup and navigation of the site; (2) ease of learning: how fast a user can learn to accomplish basic tasks on the site; (3) efficiency of use: how fast a user can accomplish basic tasks for the site; (4) memorability: the ability of the user to remember the site in order to effectively use in in future visits; (5) error frequency and severity: how often users make errors when using the site, the severity of the errors, and how users recover from the errors; and (6) satisfaction: the users’ feelings and opinions about the website. Two additional dimensions were assessed: (7) usefulness and (8) visual appeal. These usability testing procedures are consistent with best practices and procedures used for other popular tobacco cessation websites (eg, Smokefree.gov) [53].

### Data Analysis

The audio recordings from the usability sessions were transcribed. Selective coding [58] was used to identify participant comments on chosen areas of usability: intuitive design, ease of learning, efficiency of use, memorability, error frequency and severity, satisfaction, usefulness, and visual appeal. Selective coding involves identifying central categories and other categories that are systematically linked to it, producing a coherent explanation of the phenomenon under study. Structured codes were developed to guide the selective coding process. These codes were iteratively refined through team discussions and consensus coding, ensuring consistent application of codes across the dataset. Two members of the research team (MK and AD) reviewed the transcripts. An iterative process was used to map interview comments onto each usability area. Discrepancies between reviewers were resolved through discussion. Notes were used to clarify any potentially confusing content from transcripts. In addition, notes made about participants’ performance during usability testing, including types of errors made and requests for help, were used to identify usability issues.

### Ethical Considerations

Ethical approval for this study (#1598749) was obtained from the Institutional Review Board of the VA Bedford Healthcare System, Bedford, Massachusetts. Participants were US veterans recruited from the Tobacco Cessation Program and from flyers at the VA Bedford Healthcare System in Bedford, Massachusetts. There was a complete discussion of the study with potential participants, and all participants provided written informed consent to participate in the study. Veterans were informed that refusal to participate will be accepted without hesitation at any time and would not change their eligibility for VA services, treatment, disability payments, or other related VA benefits. To protect participants’ confidentiality, identifiable information collected as part of the study screening and enrollment procedures was stored in a VA shared drive that was not accessible to anyone outside the research team, and each participant was assigned a code that linked all of their data. The participants were compensated up to US $100 to complete all study assessments and usability testing. Compensation was not linked to the completion of study activities. 

## Results

### Sample Description

Veterans were aged an average of 52.4 (SD 12.3) years. They reported smoking for 28.7 (SD 13.4) years on average, with smoking an average of 11.1 (SD 4.5) cigarettes per day. Additional demographic information is provided below (Table 2).

**Table 2:** Demographic characteristics of study participants (N=16).

Sample characteristics		Values, n (%)
**Gender, n (%)**		
	Men	10 (62.5)
	Women	6 (37.5)
**Race, n (%)**		
	White	12 (75)
	Black	4 (25)
	Asian	0 (0)
	Native Hawaiian or Pacific Islander	0 (0)
	American Indian or Alaska Native	0 (0)
**Ethnicity, n (%)**		
	Non-Hispanic or Latino	16 (100)
	Hispanic or Latino	0 (0)
**Mental health diagnoses^a^, n (%)**		
	Depressive disorders	14 (87.5)
	PTSD^b^	13 (81.3)
	Anxiety disorders	7 (43.8)
	Bipolar disorder	1 (6.3)
	Schizophrenia and other psychotic spectrum disorders	1 (6.3)

^a^Categories not mutually exclusive and may not equal 100%.

^b^PTSD: posttraumatic stress disorder.

### Qualitative Feedback: Usability Areas

#### Intuitive Design

Most participants indicated that the Vet WebQuit layout was easy to navigate and use (n=15, 94%). They stated that they liked the structure, which was self-explanatory and user-friendly. Participants reported liking how the program explained material and that they could find what they needed. Participants indicated that they liked the step structure of the program and the ability to access the anytime tools at the top of the program.

Takes you through the steps, step at a time -- finds things to motivate you, get support, place to go.

Participant 1

It was very easy. When I first saw the page and read across, liked how ‘my exercises’ was next to ‘home.’

Participant 13

Some participants also highlighted a few features that were less intuitive. One veteran indicated that he did not like how the program initially did not include lines or another color to distinguish hyperlinks, so he did not realize that there was additional material that could be helpful to him. Similarly, it was noted by one veteran that audio mindfulness exercises should be highlighted to allow users to understand that they should be clicked on to be used and to provide information on how long these exercises are before they start them.

Make links red. They are important and are getting swallowed by the black. Wanted to click on them but didn't realize they were hyperlinks.

Participant 10

Point to the audio exercise! Decorate the audio exercise. Give people a heads up that it is six minutes.

Participant 16

Two veterans also indicated that they preferred more exercises with less information and less scrolling, so that the material was not overwhelming.

Is there any way to condense all that information? Right now, no mechanism for it. It's informative. People will scroll up and down and take what they need. They can always come back to it.

Participant 10

Some pages are colorful and attention-grabbing but need not a huge scroll down of words. Finish reading one section and click next.

Participant 20

#### Ease of Learning

Two veterans indicated that they were not as tech-savvy, particularly as older veterans, and that working with a web-based program made it more difficult for them.

I’m not a computer guy. People who like computers would learn more. I just don’t like computers.

Participant 7

A lot of older people won’t be able to run this website. I can see some older friends say, “F this” and get out of the website completely. For the younger generation, it’s pretty easy.

Participant 10

However, Participant 10 indicated that even though he was not as tech-savvy, the program was easy to learn.

Once you figure it out, it’s pretty easy.

Participant 10

One veteran reported feeling unsure of what the next steps were for the program, which interfered with their ability to learn more about how the program worked.

I am a little confused by the 1-3 days for each step. I need to keep moving to figure out how it works.

Participant 20

#### Efficiency of Use

Two veterans indicated that they could move through tasks quickly, highlighting the quick login and fast-paced nature of the program.

Short login.

Participant 1

I didn’t have anything in if I didn’t want to. It was click to play. There wasn’t too much stuff. Not a huge commitment.

Participant 8

However, Participant 8 also indicated that he did not want any login to make the program even quicker.

I need something that doesn’t involve logging in. I need something quick and easy that I can hit in the moment. If I have to invest a lot of time, I won’t do it.

Participant 8

These 2 veterans indicated that they also liked how concise the information was in the program.

The quit plan – it was simple. The questions weren’t repetitive, they were to the point.

Participant 1

Nice to have short sections and click to play.

Participant 8

#### Memorability

Three veterans indicated that the program captured their attention so that they would remember to use it effectively in future visits.

The program piques my curiosity and learning. I want to read on.

Participant 3

The way it is formatted – it keeps my attention. It makes me want to learn more. It keeps me wanting to learn.

Participant 4

#### Error Frequency and Severity

Two veterans (Participants 1 and 13) encountered server errors when they were going through the program, including one time after finishing the quit plan and one time after a veteran clicked on a response. In both instances, veterans were able to move through the program.

Two veterans (Participants 9 and 19) had difficulty logging into the program. One instance was because of mistyping the domain name, and another instance included difficulties getting the program web page up. In both instances, the veterans were still able to able to login in and progress through the remaining areas of the program.

#### Overall Satisfaction

All veterans (n=16, 100%) reported finding the information in the program to be helpful.

A lot of things in the website give me hope. This is a different website than anything I have seen before. Not just about urges, but letting them go is so different.

Participant 13

Veterans were particularly satisfied with certain content in the program, including internal vs external triggers, the serenity prayer, and mindfulness.

I think it’s good. It is steering people to do more inside work. That’s a good thing.

Participant 4

The serenity prayer is more for alcoholics, but smoking – I like it.

Participant 17

I like the serenity prayer. It’s filled with wisdom.

Participant 20

It’s perfect. Reading this is an hour’s worth of therapy. It took me 2 minutes to read it. Put me in a mindful spot.

Participant 17

Overall, all veterans (n=16, 100%) reported that they liked the program and that they planned to use it.

One of the most motivating websites for quitting smoking. I want to get into it. I will definitely use this.

Participant 4

I like it just the way it is, especially the reasons to quit and the good stuff. Secondhand smoke too – how it affects my family. Being African American, it affects us even more.

Participant 11

#### Usefulness

Most veterans (n=14, 88%) indicated that the program was useful to them as a tool to quit smoking.

I don’t have to make an appointment to see a doctor. This is right at my fingertips. It’s great for introverts or people who don’t like people.

Participant 10

At one point during the survey, I was thinking about having a cigarette. I came from outside to get it accomplished. The craving subsided. Reading all the material, I got caught up. So, it changed my thought process.

Participant 12

I like interacting with the Vet WebQuit plan and part of the study. I’ve had some impulses to smoke, so this can help to stay quit.

Participant 13

I would use it anytime. I would come back because it’s informative and everything is at your fingertips.

Participant 20

Veterans reported that several features of the program were useful. Specific examples of features that were mentioned as helpful included the quit plan, identifying triggers, dealing with anger, preventing relapse, mindfulness, urge surfing, involving others in their quit plan, anytime tools, and information on the physical and health effects of smoking were very helpful to them. Veterans also indicated that they liked having other resources embedded in the program.

#### Visual Appeal

Most veterans (n=14, 88%) reported liking the overall layout.

Simple. Not complicated and not busy.

Participant 1

I like that it is lowkey and not jumping at me too much. It is really focused on tracking and progress. Nice scenery, friendly, organized, but laidback.

Participant 6

Veterans had mixed reactions about the font and spacing included in the program. Two veterans reported difficulties with these elements when viewed on a mobile phone.

I think to begin with, that font can be a tiny bit bigger. Shorten the space at the top if you can. A lot of people will have trouble. I tried in horizontal and didn’t like it.

Participant 10

Some was fine print and italic that was small on the phone screen.

Participant 17

Three veterans reported that the font size was fine.

Spaced well. Good sized text.

Participant 1

I like that the writing is big.

Participant 6

Fonts are fine.

Participant 20

Several veterans (n=6, 38%) specifically reported that they liked the colors of the program, which were based in the VA style guide.

Color is important. Looking at positivity, colors are lowkey, pretty, subdued.

Participant 6

I like the colors, pictures, layout, everything. I want to congratulate the team that put this together.

Participant 11

However, 2 veterans didn’t like the darker images that were included.

Pictures that are darker ones are ominous. Stay clear of doom and gloom.

Participant 1

I don’t like the picture of water – it looks like everything is gray, so it doesn’t standout.

Participant 9

Most veterans (n=12, 75%) also reported liking the pictures that were selected, including the inspirational, friendly, and relaxing characteristics of the images.

Friendly pictures.

Participant 6

Did a good job. Each picture goes with each resource.

Participant 7

The pictures were vibrant.

Participant 9

Several veterans (n=7, 44%) specifically reported that they felt like the program was veteran-centric.

Photos are awesome. Vet forward. Red, white, and blue.

Participant 3

Veteran-sensitive. A lot of veterans would benefit from it, especially those with PTSD. Looks good.

Participant 4

I think it is very veteran-centric. You don’t really see people in uniform. That doesn’t matter.

Participant 8

#### Participant Recommendations

Veterans mentioned specific recommendations for the program, including wanting more information on the health aspects of smoking as a way to motivate them to quit smoking. Specifically, they were concerned about their breathing and lung capacity, and how that was affected by smoking. Similarly, veterans were interested in more facts about the effects of smoking (eg, nicotine) and were interested in including them in the program. In addition, they suggested mentioning specific mental health concerns that could be targeted in the program since they served as triggers for smoking, including nightmares, boredom, and social isolation.

## Discussion

### Principal Results

Usability testing is an important process in digital intervention development, since it can assess how well users can perform needed tasks in the intervention. In this study, we interviewed participants to understand their reactions to the Vet WebQuit intervention, how well they could navigate and use the web-based program, and their satisfaction with the content and design. Overall, veterans found the Vet WebQuit layout was simple and easy to navigate and use. This feedback is consistent with other digital intervention research for veterans with mental health disorders that indicates that they prefer simple and straightforward digital intervention designs [59]. Furthermore, individuals with SMI have also reported that if digital interventions are not simple to use, they experience increased stress [60], reinforcing the need to ensure that digital smoking cessation interventions have simple layouts that are easy to navigate. Supporting this, veterans liked having both the content of the program clearly broken down into steps that they could progress through, as well as content that they could easily access at any time (ie, anytime tools). They preferred features in the program that could help them to navigate (eg, audio buttons and clearly distinguished hyperlinks). They also preferred having succinct content that they could easily understand and digest.

Since most veterans who smoke and use VA tobacco cessation services are older [61], and this is reflected in our sample, we received several comments on concerns about computer literacy among older veterans and being able to use the program. However, most veterans who used the program found that they were easily able to use the program and learn how to use the features. This supports previous research on WebQuit showing that older adults in the general population were more engaged with the program and quit smoking at the same rate as middle-aged and younger adults [62], which is contrary to the popular myth that older adults have difficulty with technology [63]. If veterans encountered errors, they mostly found them in logging in to the program, highlighting the need to make the program as easy to access as possible. However, they did prefer knowing what the next steps for the program were going to be, so having that clearly laid out for them ahead of time was a preferred feature.

The present sample of veterans also reported liking having an easily accessible program that they could use at any time of the day, since they could easily log in to read over information and get help for cravings as they occurred in their day-to-day life. Since few veterans with mental health disorders engage in tobacco cessation counseling in a given year [6%, [61]], having an accessible tobacco cessation program is important to increase the chances that veterans with mental health disorders will engage in care. Furthermore, veterans with mental health disorders often face other challenges to accessing health care services (eg, transportation, remembering appointments, and responding to personal crises) [64]. In addition, often services for tobacco cessation will be offered weekly or less, and having a web-based tobacco cessation program at their “fingertips” was a widely reported positive aspect of the self-management–focused Vet WebQuit program. However, although the Vet WebQuit program is designed as a self-management digital intervention program, veterans indicated that they also liked having information on other resources that they could use for more support, such as information on counseling programs. This supports our other study on digital interventions for tobacco cessation for veterans, where veterans reported wanting to have a digital self-management program, but with someone they could also talk to for additional support [65]. Having clinician support can also boost the low rates of use of digital interventions for veterans [59]. Future work on self-management tobacco cessation programs for veterans would benefit from evaluating the use and acceptability of clinician-supported digital interventions and the best models for implementing this type of care in VA health care settings.

This sample of veterans reported that several features of the program were useful, including the quit plan, identification of triggers, content that targets mental health concerns (eg, dealing with anger), information on the health effects of smoking, tools for managing triggers (eg, mindfulness and urge surfing), and involving others in their quit plan. More information is needed, using a broader sample of veterans with mental health disorders, on the usefulness of these and other features of Vet WebQuit.

These veterans also reported that particular features of the ACT approach for tobacco cessation were appealing to them. They reported liking the distinction in the program between internal and external triggers. Consistent with this, they mentioned liking the inclusion of the serenity prayer, which helps users to understand how to manage internal triggers (“grant me the serenity to accept the things I cannot change”) and external triggers (“courage to change the things I can”) and the importance of distinguishing between these 2 types of triggers (“and the wisdom to know the difference”). They also reported especially liking the mindfulness exercises, which they could use as a tool to help them manage their cravings by not pushing the craving away but acknowledging it, and then putting their attention to something else, thereby reducing the intensity of the craving. This finding is consistent with other literature that shows that users of digital smoking cessation interventions prefer having information in these interventions on how to cope with negative emotions like anxiety [66]. However, these needs are even more heightened for veterans with mental health disorders since they are coping with more frequent and intense emotions that trigger smoking [6]. Therefore, having increased and detailed ACT-focused content on how to manage emotions such as anger and anxiety using acceptance and mindfulness, and making the distinction between internal triggers and external triggers, provides important guidance that veterans with mental health disorders find particularly useful. Overall, ACT digital interventions for smoking cessation show high satisfaction, user engagement, and efficacy for people with mental disorders [67].

Finally, participants recommended including more information on the health aspects of smoking. This recommendation is in line with more general feedback from users of smoking cessation digital interventions, with users indicating that they want to have information on what they could gain and lose through smoking cessation [68].

### Limitations

This study had several strengths and limitations. A strength of this study was the qualitative approach, with a more intensive “think-aloud” procedure that had veterans work with the program and provide feedback in the moment as they used it. Using qualitative data to understand how to make digital health interventions more acceptable to the user is a core process in the development of these programs.

The limitations of this study include the small sample of 16 veterans from one medical center. There may be varying feedback depending on the geographical location, digital literacy, and demographic characteristics of veterans (eg, rurality, race, and ethnicity). For instance, obtaining feedback from more rural users may be helpful to determine if the acceptability is the same for people who are not as close to VA health care locations and have fewer resources in the community. Therefore, the conclusions of this particular study should be tempered. In addition, this study did not focus on evaluating the effectiveness of the intervention, only on usability and acceptability. Future research is needed to understand the effectiveness of the Vet WebQuit intervention for veterans with mental health disorders.

### Conclusions

Overall, veterans with mental health disorders are in need of accessible and engaging digital tobacco cessation interventions. Veterans with mental health disorders found that the Vet WebQuit program was easy to navigate and use, and liked particular features, such as the quit plan, identification of triggers, targeted mental health content, information on the health effects of smoking, and ACT-focused skills and strategies for managing smoking triggers (eg, internal vs external triggers and mindfulness exercises). Overall, this study demonstrated that Vet WebQuit was acceptable to veterans and they found it useful. Future research should focus on the effectiveness of this program for veterans with mental health disorders. However, given the positive responses to the Vet WebQuit program, our study demonstrates that this program may fill an important gap in accessible smoking cessation services targeted at mental health obstacles to quitting smoking.

## Data Availability

The datasets generated and analyzed during this study are not publicly available due to the security requirements of the Department of Veterans Affairs, but are available from the corresponding author on reasonable request. The authors will consider reasonable requests on a case-by-case basis, subject to compliance with the Department of Veterans Affairs data sharing agreements.

## Abbreviations

ACT: acceptance and commitment therapy
CBT: cognitive behavioral therapy
DSM-5: Diagnostic and Statistical Manual of Mental Disorders (Fifth Edition)
PTSD: posttraumatic stress disorder
SCID-5-RV: Structured Clinical Interview for DSM-5 Research Version
SMI: serious mental illness
VA: Veterans Affairs
VHA: Veterans Healthcare Administration

## References

[1] Odani S, Agaku IT, Graffunder CM, Tynan MA, Armour BS (2018). Tobacco Product Use Among Military Veterans - United States, 2010-2015. MMWR Morb Mortal Wkly Rep.

[2] Lasser K, Boyd JW, Woolhandler S, Himmelstein DU, McCormick D, Bor DH (2000). Smoking and mental illness: A population-based prevalence study. JAMA.

[3] CDC (2013). Current cigarette smoking among adults—United States, 2011. JAMA.

[4] Prochaska JJ, Das S, Young-Wolff KC (2017). Smoking, Mental Illness, and Public Health. Annu Rev Public Health.

[5] McFall M, Saxon AJ, Thompson CE, Yoshimoto D, Malte C, Straits-Troster K, Kanter E, Zhou XHA, Dougherty CM, Steele B (2005). Improving the rates of quitting smoking for veterans with posttraumatic stress disorder. Am J Psychiatry.

[6] Piper ME, Smith SS, Schlam TR, Fleming MF, Bittrich AA, Brown JL, et al (2010). Psychiatric disorders in smokers seeking treatment for tobacco dependence: relations with tobacco dependence and cessation. J Consult Clin Psychol.

[7] Ziedonis D, Hitsman B, Beckham JC, Zvolensky M, Adler LE, Audrain-McGovern J, Breslau N, Brown RA, George TP, Williams J, Calhoun PS, Riley WT (2008). Tobacco use and cessation in psychiatric disorders: National Institute of Mental Health report. Nicotine Tob Res.

[8] (2014). The health consequences of smoking - 50 years of progress: a report of the Surgeon General. National Center for Chronic Disease Prevention and Health Promotion (US) Office on Smoking and Health.

[9] Colton CW, Manderscheid RW (2006). Congruencies in increased mortality rates, years of potential life lost, and causes of death among public mental health clients in eight states. Prev Chronic Dis.

[10] Beckham J, Wiley M, Miller S, Dennis M, Wilson S, McClernon FJ, Calhoun PS (2008). Ad lib smoking in post-traumatic stress disorder: an electronic diary study. Nicotine Tob Res.

[11] Feldner MT, Babson KA, Zvolensky MJ (2007). Smoking, traumatic event exposure, and post-traumatic stress: a critical review of the empirical literature. Clin Psychol Rev.

[12] Kastaun S, Brose LS, Scholz E, Viechtbauer W, Kotz D (2022). Mental Health Symptoms and Associations with Tobacco Smoking, Dependence, Motivation, and Attempts to Quit: Findings from a Population Survey in Germany (DEBRA Study). Eur Addict Res.

[13] Keller-Hamilton B, Moe AM, Breitborde NJK, Lee A, Ferketich AK (2019). Reasons for smoking and barriers to cessation among adults with serious mental illness: A qualitative study. J Community Psychol.

[14] Trainor K, Leavey G (2017). Barriers and Facilitators to Smoking Cessation Among People With Severe Mental Illness: A Critical Appraisal of Qualitative Studies. Nicotine Tob Res.

[15] Fu S, McFall M, Saxon A, Beckham J, Carmody T, Baker D, Joseph AM (2007). Post-traumatic stress disorder and smoking: a systematic review. Nicotine Tob Res.

[16] Iasevoli F, Balletta R, Gilardi V, Giordano S, de Bartolomeis A (2013). Tobacco smoking in treatment-resistant schizophrenia patients is associated with impaired cognitive functioning, more severe negative symptoms, and poorer social adjustment. Neuropsychiatr Dis Treat.

[17] Depp CA, Bowie CR, Mausbach BT, Wolyniec P, Thornquist MH, Luke JR, McGrath JA, Pulver AE, Patterson TL, Harvey PD (2015). Current smoking is associated with worse cognitive and adaptive functioning in serious mental illness. Acta Psychiatr Scand.

[18] Berk M, Ng F, Wang WV, Tohen M, Lubman DI, Vieta E, Dodd S (2008). Going up in smoke: tobacco smoking is associated with worse treatment outcomes in mania. J Affect Disord.

[19] Satre DD, Kohn CS, Weisner C (2007). Cigarette smoking and long-term alcohol and drug treatment outcomes: a telephone follow-up at five years. Am J Addict.

[20] Mahoney CT, Zweig IR, Marx BP, Keane TM (2020). Cross-lagged effects of posttraumatic stress disorder symptom severity and cigarette smoking among OEF/OIF/OND veterans. Depress Anxiety.

[21] Vidrine JI, Cofta-Woerpel L, Daza P, Wright KL, Wetter DW (2006). Smoking cessation 2: behavioral treatments. Behav Med.

[22] McFall M, Saxon AJ, Malte CA, Chow B, Bailey S, Baker DG, Beckham Jean C, Boardman Kathy D, Carmody Timothy P, Joseph Anne M, Smith Mark W, Shih Mei-Chiung, Lu Ying, Holodniy Mark, Lavori Philip W, CSP 519 Study Team (2010). Integrating tobacco cessation into mental health care for posttraumatic stress disorder: a randomized controlled trial. JAMA.

[23] Feldner MT, Smith RC, Monson CM, Zvolensky MJ (2013). Initial evaluation of an integrated treatment for comorbid PTSD and smoking using a nonconcurrent, multiple-baseline design. Behav Ther.

[24] Hayes SC, Luoma JB, Bond FW, Masuda A, Lillis J (2006). Acceptance and commitment therapy: model, processes and outcomes. Behav Res Ther.

[25] McClure JB, Bricker J, Mull K, Heffner JL (2020). Comparative Effectiveness of Group-Delivered Acceptance and Commitment Therapy versus Cognitive Behavioral Therapy for Smoking Cessation: A Randomized Controlled Trial. Nicotine Tob Res.

[26] Bricker JB, Mull KE, Kientz JA, Vilardaga R, Mercer LD, Akioka KJ, Heffner JL (2014). Randomized, controlled pilot trial of a smartphone app for smoking cessation using acceptance and commitment therapy. Drug Alcohol Depend.

[27] Bricker JB (2011). Acceptance and commitment therapy: a promising approach to smoking cessation. Mindfulness and Acceptance in Behavioral Medicine: Current Theory and Practice.

[28] Bricker JB, Bush T, Zbikowski SM, Mercer LD, Heffner JL (2014). Randomized trial of telephone-delivered acceptance and commitment therapy versus cognitive behavioral therapy for smoking cessation: a pilot study. Nicotine Tob Res.

[29] Gifford EV, Kohlenberg BS, Hayes SC, Antonuccio DO, Piasecki MM, Rasmussen-Hall ML, Palm KM (2004). Acceptance-based treatment for smoking cessation. Behavior Therapy.

[30] Gifford EV, Kohlenberg BS, Hayes SC, Pierson HM, Piasecki MP, Antonuccio DO, Palm KM (2011). Does acceptance and relationship focused behavior therapy contribute to bupropion outcomes? A randomized controlled trial of functional analytic psychotherapy and acceptance and commitment therapy for smoking cessation. Behav Ther.

[31] Hernández-López Mónica, Luciano MC, Bricker JB, Roales-Nieto JG, Montesinos F (2009). Acceptance and commitment therapy for smoking cessation: a preliminary study of its effectiveness in comparison with cognitive behavioral therapy. Psychol Addict Behav.

[32] Kelly MM, Latta RE, Gimmestad K (2012). Acceptance and Mindfulness-Based Tobacco Cessation Interventions for Individuals With Mental Health Disorders. Journal of Dual Diagnosis.

[33] Bach P, Hayes SC, Gallop R (2012). Long-term effects of brief acceptance and commitment therapy for psychosis. Behav Modif.

[34] Hayes SC (2016). Acceptance and Commitment Therapy, Relational Frame Theory, and the Third Wave of Behavioral and Cognitive Therapies - Republished Article. Behav Ther.

[35] Hayes SC, Levin ME, Plumb-Vilardaga J, Villatte JL, Pistorello J (2013). Acceptance and commitment therapy and contextual behavioral science: examining the progress of a distinctive model of behavioral and cognitive therapy. Behav Ther.

[36] Shawyer F, Farhall J, Thomas N, Hayes SC, Gallop R, Copolov D, Castle DJ (2017). Acceptance and commitment therapy for psychosis: randomised controlled trial. Br J Psychiatry.

[37] Thomas N, Shawyer F, Castle DJ, Copolov D, Hayes SC, Farhall J (2014). A randomised controlled trial of acceptance and commitment therapy (ACT) for psychosis: study protocol. BMC Psychiatry.

[38] Record EJ, Medoff DR, Dixon LB, Klingaman EA, Park SG, Hack S, Brown CH, Fang LJ, Kreyenbuhl J (2016). Access to and Use of the Internet by Veterans with Serious Mental Illness. Community Ment Health J.

[39] Bricker JB (2017). How contextual behaviorism can impact public health: results on the largest clinical trial of ACT. https://contextualscience.org/sites/default/files/wc15%20whole%20program%206.2%20web.pdf?utm.

[40] Bricker J, Wyszynski C, Comstock B, Heffner JL (2013). Pilot randomized controlled trial of web-based acceptance and commitment therapy for smoking cessation. Nicotine Tob Res.

[41] Gierisch J, Straits-Tröster K, Calhoun P, Beckham JC, Acheson S, Hamlett-Berry K (2012). Tobacco use among Iraq- and Afghanistan-era veterans: a qualitative study of barriers, facilitators, and treatment preferences. Prev Chronic Dis.

[42] Michie S, Yardley L, West R, Patrick K, Greaves F (2017). Developing and Evaluating Digital Interventions to Promote Behavior Change in Health and Health Care: Recommendations Resulting From an International Workshop. J Med Internet Res.

[43] Klee A, Stacy M, Rosenheck R, Harkness L, Tsai J (2016). Interest in technology-based therapies hampered by access: A survey of veterans with serious mental illnesses. Psychiatr Rehabil J.

[44] Tsai J, Klee A, Rosenheck RA, Harkness L (2014). Internet use among veterans with severe mental illness. Psychiatr Serv.

[45] Allen RS, Guadagno RE, Parmelee P, Minney JA, Hilgeman MM, Tabb KD, McNeil SF, Houston T, Kertesz S, Davis L (2013). Internet connectivity among rural Alabama veterans: baseline findings from the Alabama Veterans Rural Health Initiative Project. Rural Remote Health.

[46] Kelly MM, Heffner JL, Mull KE, Bricker JB (2016). Receptivity of a web-delivered ACT smoking cessation treatment for smokers with posttraumatic stress disorder symptoms. https://contextualscience.org/wc14_symposia_detail.

[47] Weinschenk S Usability: A Business Case.

[48] Cheh JA, Ribisl KM, Wildemuth BM (2003). An assessment of the quality and usability of smoking cessation information on the Internet. Health Promot Pract.

[49] Brunette MF, Ferron JC, Devitt T, Geiger P, Martin WM, Pratt S, Santos M, McHugo GJ (2012). Do smoking cessation websites meet the needs of smokers with severe mental illnesses?. Health Educ Res.

[50] Robinson LJ, Thompson JM, Gallagher P, Goswami U, Young AH, Ferrier IN, Moore PB (2006). A meta-analysis of cognitive deficits in euthymic patients with bipolar disorder. J Affect Disord.

[51] Mur M, Portella MJ, Martínez-Arán Anabel, Pifarré Josep, Vieta E (2007). Persistent neuropsychological deficit in euthymic bipolar patients: executive function as a core deficit. J Clin Psychiatry.

[52] Chaves OC, Lombardo LE, Bearden CE, Woolsey MD, Martinez DM, Barrett JA, Miller AL, Velligan DI, Glahn DC (2011). Association of clinical symptoms and neurocognitive performance in bipolar disorder: a longitudinal study. Bipolar Disord.

[53] Stoddard JL, Augustson EM, Mabry PL (2006). The importance of usability testing in the development of an internet-based smoking cessation treatment resource. Nicotine Tob Res.

[54] First MB, Williams JB, Karg RS, Spitzer RL (2015). Structured clinical interview for DSM-5 disorders-research version (SCID-5 for DSM-5, Research Version; SCID-5-RV).

[55] Brown RA, Lejuez CW, Kahler CW, Strong DR (2002). Distress tolerance and duration of past smoking cessation attempts. Journal of Abnormal Psychology.

[56] Hwang W, Salvendy G (2010). Number of people required for usability evaluation. Commun. ACM.

[57] Usability. Digital.gov.

[58] Strauss AL, Corbin J (1998). Basics of Qualitative Research: Techniques and Procedures for Developing Grounded Theory.

[59] Buck B, Nguyen J, Porter S, Ben-Zeev D, Reger GM (2022). FOCUS mHealth Intervention for Veterans With Serious Mental Illness in an Outpatient Department of Veterans Affairs Setting: Feasibility, Acceptability, and Usability Study. JMIR Ment Health.

[60] Vilardaga R, Rizo J, Ries R, Kientz J, Ziedonis D, Hernandez K, McClernon Francis J (2019). Formative, multimethod case studies of learn to quit, an acceptance and commitment therapy smoking cessation app designed for people with serious mental illness. Transl Behav Med.

[61] Kelly MM, Sido H, Rosenheck R (2016). Rates and correlates of tobacco cessation service use nationally in the Veterans Health Administration. Psychol Serv.

[62] Kwon DM, Santiago-Torres M, Mull KE, Sullivan BM, Bricker JB (2022). Older adults who smoke: Do they engage with and benefit from web-based smoking cessation interventions?. Prev Med.

[63] Wandke H, Sengpiel M, Sönksen M (2012). Myths about older people's use of information and communication technology. Gerontology.

[64] Drapalski AL, Milford J, Goldberg RW, Brown CH, Dixon LB (2008). Perceived Barriers to Medical Care and Mental Health Care Among Veterans With Serious Mental Illness. Psychiatric Services.

[65] Heffner JL, Kelly MM, Reilly ED, Reece SG, Claudio T, Serfozo E, Baker K, Watson NL, Karekla M (2023). An Avatar-Led Web-Based and SMS Text Message Smoking Cessation Program for Socioeconomically Disadvantaged Veterans: Pilot Randomized Controlled Trial. JMIR Form Res.

[66] Zhang J, Cao Y, Mo H, Feng R (2023). The association between different types of physical activity and smoking behavior. BMC Psychiatry.

[67] Santiago-Torres M, Mull KE, Sullivan BM, Prochaska JJ, Zvolensky MJ, Bricker JB (2024). Can an Acceptance and Commitment Therapy-Based Smartphone App Help Individuals with Mental Health Disorders Quit Smoking?. Depress Anxiety.

[68] Zhang M, Wolters M, O'Connor S, Wang Y, Doi L (2023). Smokers' user experience of smoking cessation apps: A systematic review. Int J Med Inform.

